# Capacitance effect on the oscillation and switching characteristics of spin torque oscillators

**DOI:** 10.1186/1556-276X-9-597

**Published:** 2014-11-03

**Authors:** Tui Zeng, Yan Zhou, Chi Wah Leung, Peter PT Lai, Philip WT Pong

**Affiliations:** 1 Department of Electrical and Electronic Engineering, The University of Hong Kong, Pokfulam, Hong Kong; 2 Department of Physics, The University of Hong Kong, Pokfulam, Hong Kong; 3 Department of Applied Physics, The Hong Kong Polytechnic University, Hung Hom, Hong Kong

**Keywords:** Capacitance effect, Oscillation frequency, Canted region, Switching time

## Abstract

We have studied the capacitance effect on the oscillation characteristics and the switching characteristics of the spin torque oscillators (STOs). We found that when the external field is applied, the STO oscillation frequency exhibits various dependences on the capacitance for injected current ranging from 8 to 20 mA. The switching characteristic is featured with the emerging of the canted region; the canted region increases with the capacitance. When the external field is absent, the STO free-layer switching time exhibits different dependences on the capacitance for different injected current. These results help to establish the foundation for capacitance-involved STO modeling.

## Background

The conventional way of changing the magnetization of a thin film is usually realized through applying an external magnetic field. In recent years, it has been found both theoretically [1-3] and experimentally [4,5] that a spin-polarized current which carries more spin up or spin down electrons can also change the magnetization when passing through the thin film. This effect helps to generate steady precession of the free-layer magnetization in a spin valve structure by an injected spin-polarized current, which results in a periodic variation of the device resistance and forms spin-torque oscillators (STOs) [6-12]. The advantages of the STO are its capability of generating microwave with ultra-wide bandwidth (from 100 MHz to 60 GHz) and its easy modulation at very high frequency. Its potential application as microwave generator has received unprecedented attention. Among the many unrevealed problems remained in the STO area, much research effort focuses on the STO authentic modeling. However, the capacitance effect is not considered at all in most previous studies [13-15]. Capacitance effect [13-15] being introduced by intrinsic sources (parasitic capacitance due to the interaction between the multilayer thin films in STOs) and extrinsic sources (lead capacitance due to the connection between the external IC and STOs) is inevitable during the preparation process of spin-torque oscillators (typically GMR multilayers). Therefore, in order to accurately reflect the characteristics of prepared spin-torque oscillator devices, it is highly essential to explore the capacitance effect on oscillation characteristics and switching characteristics. Meanwhile, this research not only helps to establish the foundation for capacitance-involved STO modeling but also helps to reveal the origin of capacitance effect in nanodevices. Since our findings could be applied in the modeling of authentic STO, which is highly beneficial for supporting and guiding the fabrication process in nanotechnology and nanoscience industry.

In this paper, a circuit model where a capacitor connected in parallel with a STO is proposed. The marcospin model is adopted to explore how the magnetodynamics of the STO is influenced by the capacitor. The oscillation characteristics and the switching characteristics are both fully studied.

## Methods

**Figure 1:**
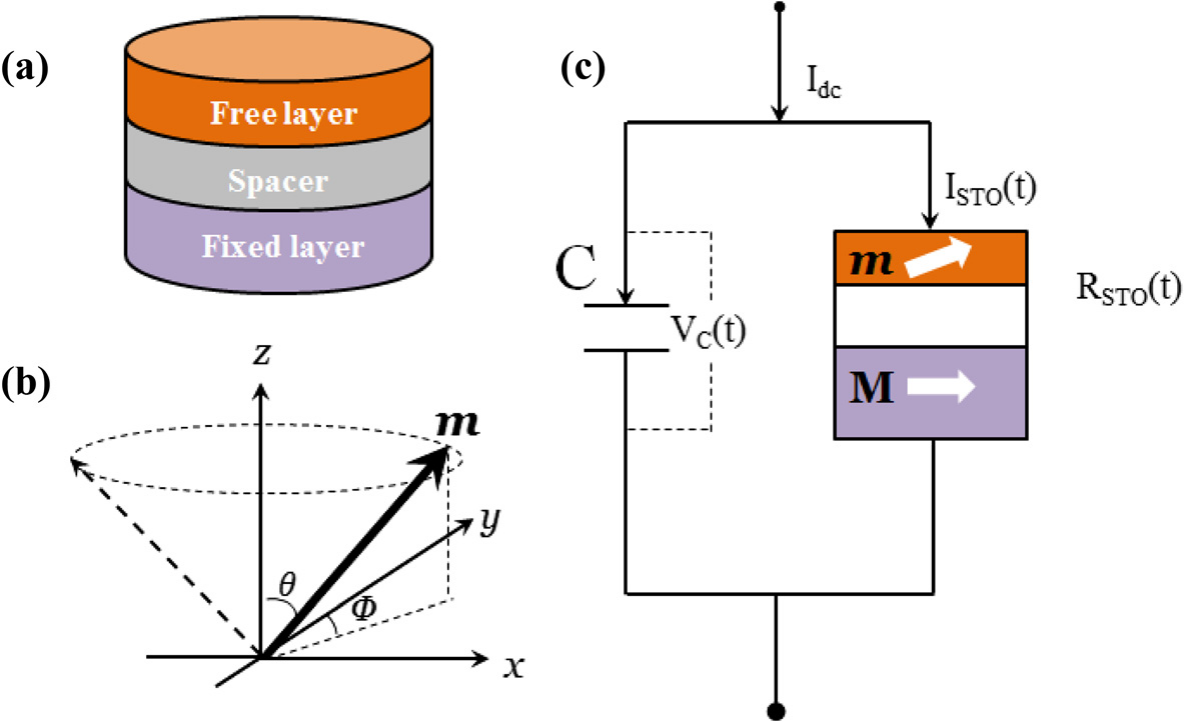
**Schematic illustration of the spin torque oscillator. (a)** Device structure. **(b)** Free-layer magnetization in spherical coordinates. **(c)** Sketch of a STO connected in parallel with a capacitor.

As shown in Figure 1, a giant magnetoresistance (GMR)-based STO consisting of a fixed layer, a nonmagnetic layer, and a free layer is modeled with a capacitor connected in parallel. An ideal current source *I*_dc_ is applied. The time evolution of the free-layer magnetization is described by the Landau-Lifshiz-Gilbert equation with Slonczewski spin torque term [2]

(1) $$\frac{d\vec{m}}{dt}=-\gamma \vec{m}\times {\vec{H}}_{\mathrm{eff}}+\alpha \vec{m}\times \frac{d\vec{m}}{dt}+\gamma \tau$$

where *m* stands for the free-layer magnetization unit vector, *γ* stands for the gyromagnetic ratio, and *α* is the Gilbert damping parameter. *H*_eff_ is the effective magnetic field acting on the free layer, and it consists of the contributions from the uniaxial magnetic anisotropy field *H*_*k*_, the demagnetization field *H*_*d*_, and the external in-plane applied magnetic field *H*_app_*.* We obtain the effective field as:

(2) $${\vec{H}}_{\mathrm{eff}}=H_{\mathrm{app}}\cdot {\vec{e}}_{x}+H_{k}\left(\vec{m}\cdot {\vec{e}}_{x}\right){\vec{e}}_{x}-H_{d}\left(\vec{m}\cdot {\vec{e}}_{z}\right){\vec{e}}_{z}$$

where *e*_*x*_ and *e*_*z*_ are the unit vectors along *x* (in-plane easy axis) and *z* (out-of-plane), respectively. In this study, the field-like spin torque term is considered [16,17]. Thus, the spin transfer torque (STT) term in Equation (1) can be written in general as:

(3) $$\tau =a_{J}\vec{m}\times \left(\vec{m}\times \vec{M}\right)+b_{J}\vec{m}\times \vec{M}$$

where *a*_*J*_ and *b*_*J*_ are the in-plane and perpendicular (or field-like) spin torque components, respectively. A linear relation between *a*_*J*_ and *b*_*J*_ is established [18] as:

(4) $$b_{J}=\beta a_{J}=\beta \cdot \left|\gamma \right|\frac{\eta I_{\mathrm{dc}}}{2\mu_{0}M_{S}eV_{f}}$$

where *μ*_0_ is the magnetic vacuum permeability, *η* is the spin transfer efficiency, *M*_*S*_ is the free-layer saturation magnetization, and V_f_ is the volume of the free layer. In this study, the free layer is composed of a typical CoFeB thin film with a circular shape with a dimension of 250 nm and thickness of 3 nm. The *b*_*J*_ term in metallic spin valve structures is small. We define |*β*| = 10% in this study. Other parameter values are presented as follows [13]: |*γ*| = 1.86 × 10^11^ Hz/T, *η* = 0.35, *M*_*S*_ = 1,270 kA/m, α = 0.008, *H*_app_ =0.05 T, *H*_*d*_ = 4π*Ms* = 1.27 T, *H*_*k*_ = 0.02 T, *R*_*P*_ = 15.8 Ω, *R*_*AP*_ = 23.4 Ω, and *e* = 1.6021764e-19.

Meanwhile, the continuity of the total dc current and the equal voltage drop across the two parallel branches result in the following equations:

(5) $$I_{\mathrm{STO}}\left(t\right)+\frac{CdV_{C}\left(t\right)}{dt}=I_{\mathrm{dc}}$$

(6) VCt=ISTOtRAP+RP−RAP−RPcosθt2

where *R*_*AP*_ and *R*_*P*_ stand for the ‘anti-parallel’ and the ‘parallel’ resistance of the STO, respectively, *θ*(*t*) is the angle between the magnetization of the fixed layer and that of the free layer, *V*_*C*_(*t*) is the instantaneous voltage across the capacitor, and *I*_*STO*_(*t*) is the current flowing through the STO branch, as shown in Figure 1c. The merging of (5) and (6) results in:

(7) $$\frac{dI_{\mathrm{STO}}}{dt}=\frac{I_{\mathrm{dc}}-I_{\mathrm{STO}}\left(t\right)-CI_{\mathrm{STO}}\left(t\right)\cdot dR\left(t\right)/dt}{CR\left(t\right)}$$

The magnetic dynamics can then be numerically solved using (1) and (7).

To further elaborate how to numerically solve Equations (1) and (7), we transform Equation (1) into the following set of differential equations in a spherical coordinate system:

(8) $$\begin{array}{ll} \frac{d\theta }{dt} & =\frac{\alpha \gamma }{1+\alpha^{2}}\left(H_{\text{theta}}+\beta \times {\mathrm{Is}}_{\text{theta}}+{\mathrm{Is}}_{\text{phi}}\right)\\ & \;+\frac{\gamma }{1+\alpha^{2}}\left(H_{\mathrm{phi}}+\beta \times {\mathrm{Is}}_{\mathrm{phi}}-{\mathrm{Is}}_{\mathrm{theta}}\right) \end{array}$$

(9) $$\begin{array}{l} \;-\frac{\gamma }{1+\alpha^{2}}\left(H_{\mathrm{theta}}+\beta \times {\mathrm{Is}}_{\mathrm{theta}}+{\mathrm{Is}}_{\mathrm{phi}}\right)\\ \frac{d\phi }{dt}=\frac{+\frac{\alpha \gamma }{1+\alpha^{2}}\left(H_{\mathrm{phi}}+\beta \times {\mathrm{Is}}_{\mathrm{phi}}-{\mathrm{Is}}_{\mathrm{theta}}\right)}{\sin\left(\theta \right)} \end{array}$$

where *H*_theta_ and *H*_phi_ stand for the effective field in a spherical coordinate system, Is_theta_ and Is_phi_ stand for the current injected into the STO in a spherical coordinate system.

Equation (7) can be transformed into the following set of differential equations in a spherical coordinate system:

(10) $$\begin{array}{l} \;I_{\text{amper}}\times \frac{\eta \hbar }{2M_{S}\times \mathrm{electron}\times V_{f}}-J_{\text{tesla}}+C_{\mathrm{ap}}\times \frac{{\mathrm{Is}}_{\mathrm{theta}}}{\sin\left(\theta \right)}\\ \frac{dI_{\text{amper}}}{dt}=\frac{\times \left(R_{\mathrm{ap}}-R_{p}\right)/2\times \left(\mathrm{sum}\left(\sin\left(\theta \right)\times \frac{d\theta }{dt}\right)\right)}{C_{ap}\times (\left(R_{\mathrm{ap}}+R_{p}\right)/2\times N-\left(R_{\mathrm{ap}}-R_{p}\right)/2\times \left(\mathrm{sum}\left(\cos\left(\theta \right)\right)\right)} \end{array}$$

where *I*_amper_ stands for the total current injected into the STO and the capacitor, Cap stands for the value of capacitance. By solving Equations (8), (9), and (10) using runge-kutta method [19], the time-varying *θ*, and *I*_amper_ are identified, where the magnetic dynamics are then obtained.

## Results and discussion

### A. Oscillation characteristics with external field

**Figure 2:**
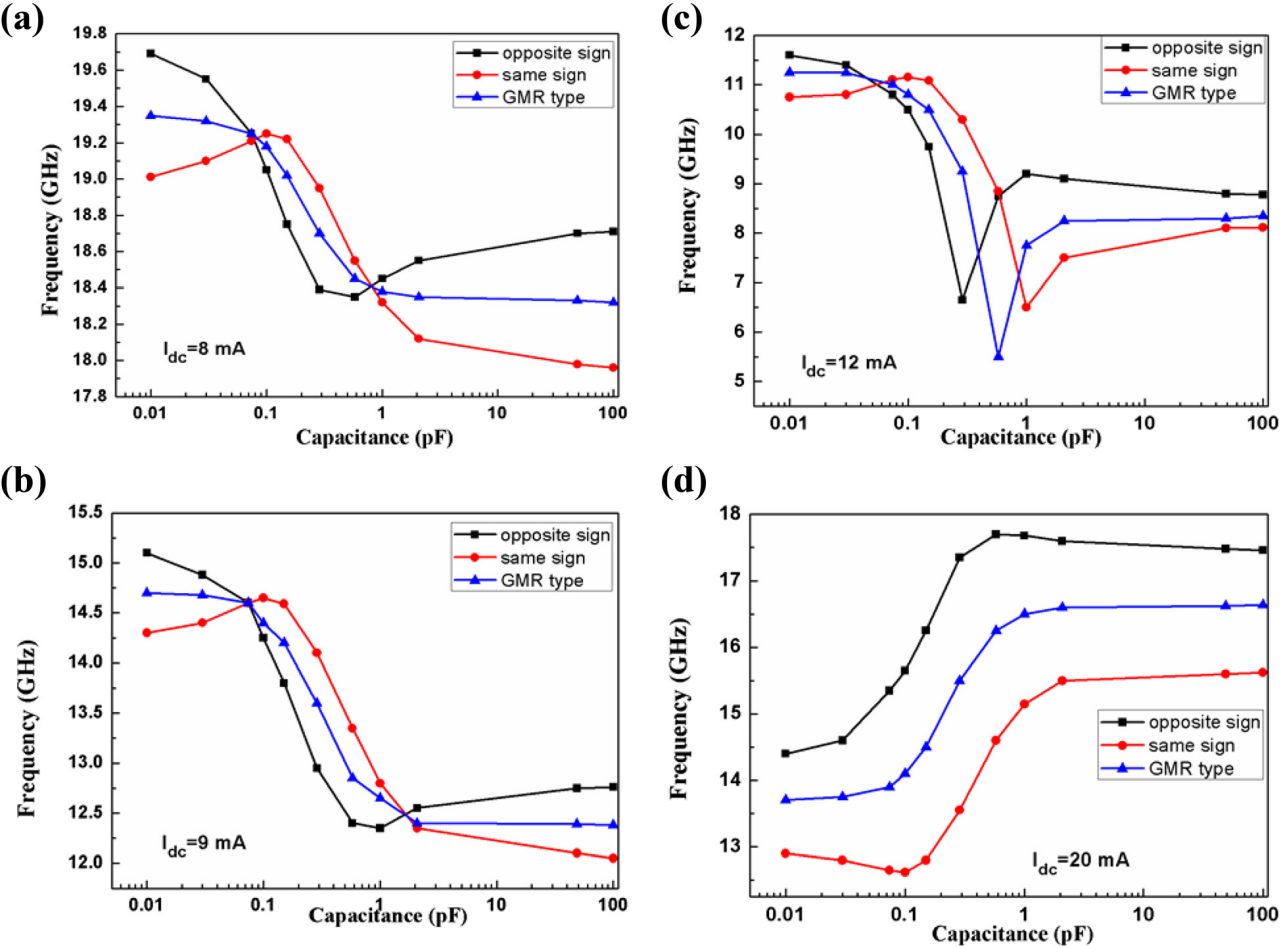
**When *H***_**app **_**= 0.05 T, the frequency dependence on capacitance when (a)***I*_dc_ = 8 mA, **(b)***I*_dc_ = 9 mA, **(c)***I*_dc_ = 12 mA, and **(d)***I*_dc_ = 20 mA.

The oscillation characteristics are studied when external field is applied along the easy axis (x-axis) with the value *H*_app_ =0.05 T. When *I*_dc_ is applied, the free layer of the STO is in a steady precessional state where a stable frequency is induced. The presence of a parallel connected capacitor shares the injected dc current with the STO, which changes the free-layer magnetization precessional state to a new orbit. The STO oscillation frequencies are presented under different capacitance values in Figure 2. ‘Opposite sign’, ‘Same sign’, and ‘GMR type’ refer to frequency vs capacitance curves when β = −10%*,* β *=*10%*,* and β =0%, respectively*.* When the injected current *I*_dc_ is relatively small (8 and 9 mA) and the field-like term is ignored (β =0%), the increase of the capacitance leads to the general decrease of the oscillation frequency, as shown in Figure 2a,b. When the capacitance is in the range of 0.01 to 0.1 pF, this negative correlation is enhanced for β = −10% whereas it changes to positive correlation for β *=*10%*.* Meanwhile, when the capacitance is in the range of 1 to 100 pF, this negative correlation is enhanced for β =10% whereas it changes to positive correlation for β *= −*10%. This phenomenon is due to the fact that the field-like term is dependent on the applied bias voltage [12,16,17]. Either relatively small capacitance value or relatively large capacitance value would result in a large change of the bias voltage, which also induce a large change of the field-like term. When *I*_dc_ reaches the value of 12 mA, a ‘V-shape’ trend formed between the frequency and the capacitance. Compared with the minimum peak for β =0%, the minimum peak for β *= −*10% occurs at lower capacitance value while it occurs at higher capacitance value for β *=*10%, as shown in Figure 2c. When *I*_dc_ reaches the value of 20 mA, general positive correlation between the capacitance and the frequency is exhibited for β = −10%*,* β *=*10%*,* and β =0%. The field-like term (either β = −10% or β *=*10%) can result in higher oscillation frequency in this case.

### B. Switching characteristics with external field

**Figure 3:**
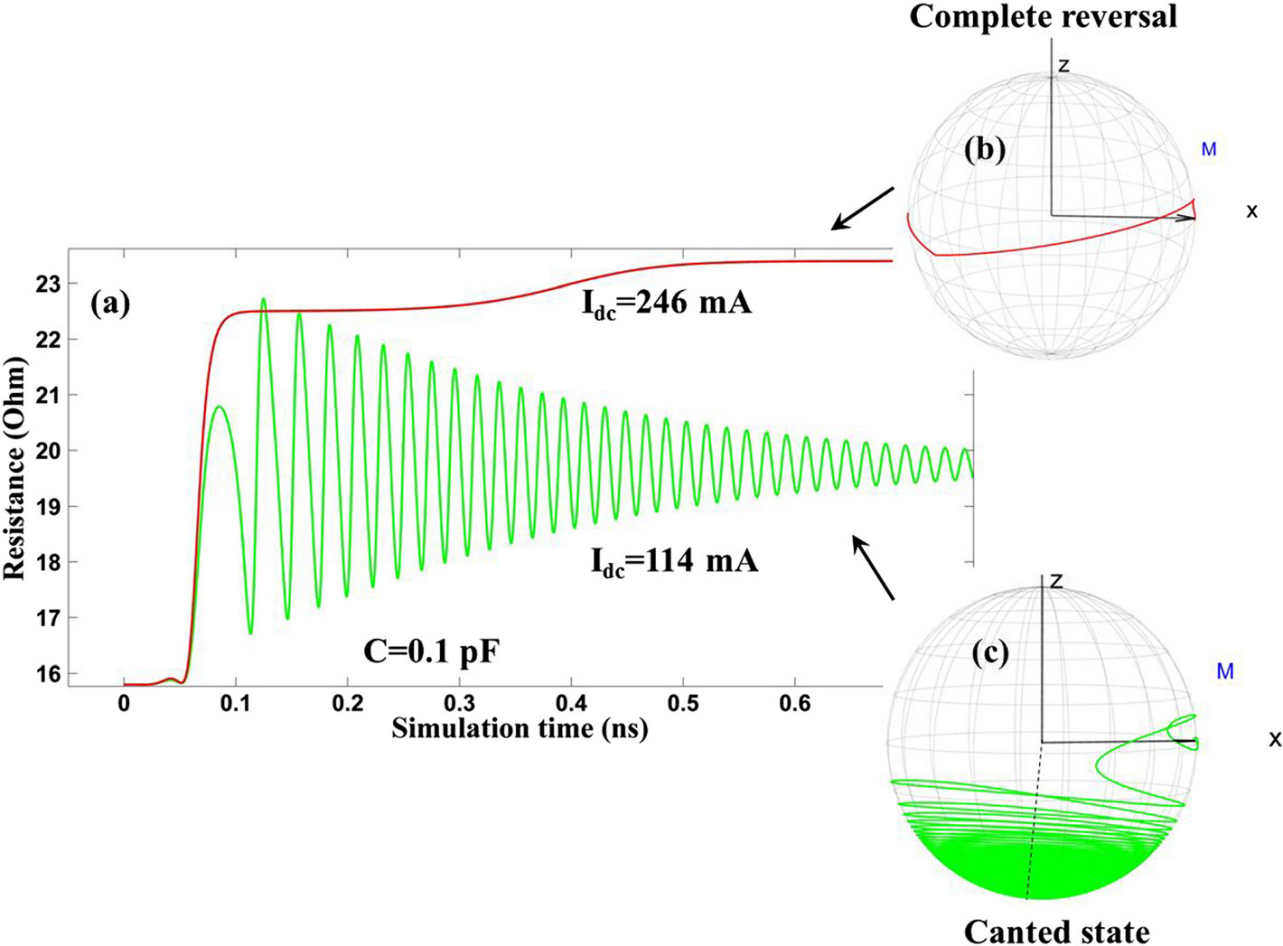
**When *H***_**app **_**= 0.05 T, *C *= 0. 1 pF. (a)** Time-evolved STO resistance for *I*_dc_ =114 mA and *I*_dc_ = 246 mA. **(b)** Free-layer magnetization switching trajectory for *I*_dc_ = 246 mA. **(c)** Free-layer magnetization switching trajectory for *I*_dc_ = 114 mA.

In Part A, it is discussed how the oscillation frequency behaves under different capacitance values. However, it is worth noting that as the injected current *I*_dc_ increases to a critical value, the balance between the injected spin torque and damping cannot be maintained. The injected spin torque overwhelms the damping, resulting in the reversal of the free-layer magnetization from parallel state to anti-parallel. However, in the case where the capacitance value is set to 0.1 pF (Figure 3), when the injected current *I*_dc_ increases gradually to 114 mA, the magnetization switches from parallel state to a canted state instead of anti-parallel state. The magnetization trajectory in Figure 3c suggests that the magnetization finally stays in a static state with a canted angle. When *I*_dc_ continues to increase to 246 mA, complete magnetization reversal is achieved from parallel state to anti-parallel state, as shown in Figure 3b. This concludes that the existence of the capacitance realizes a canted region (from 114 to 246 mA in this case) as a transition between parallel state and anti-parallel state. It has also been verified that without the capacitance, no canted region is observed in this system.

**Figure 4:**
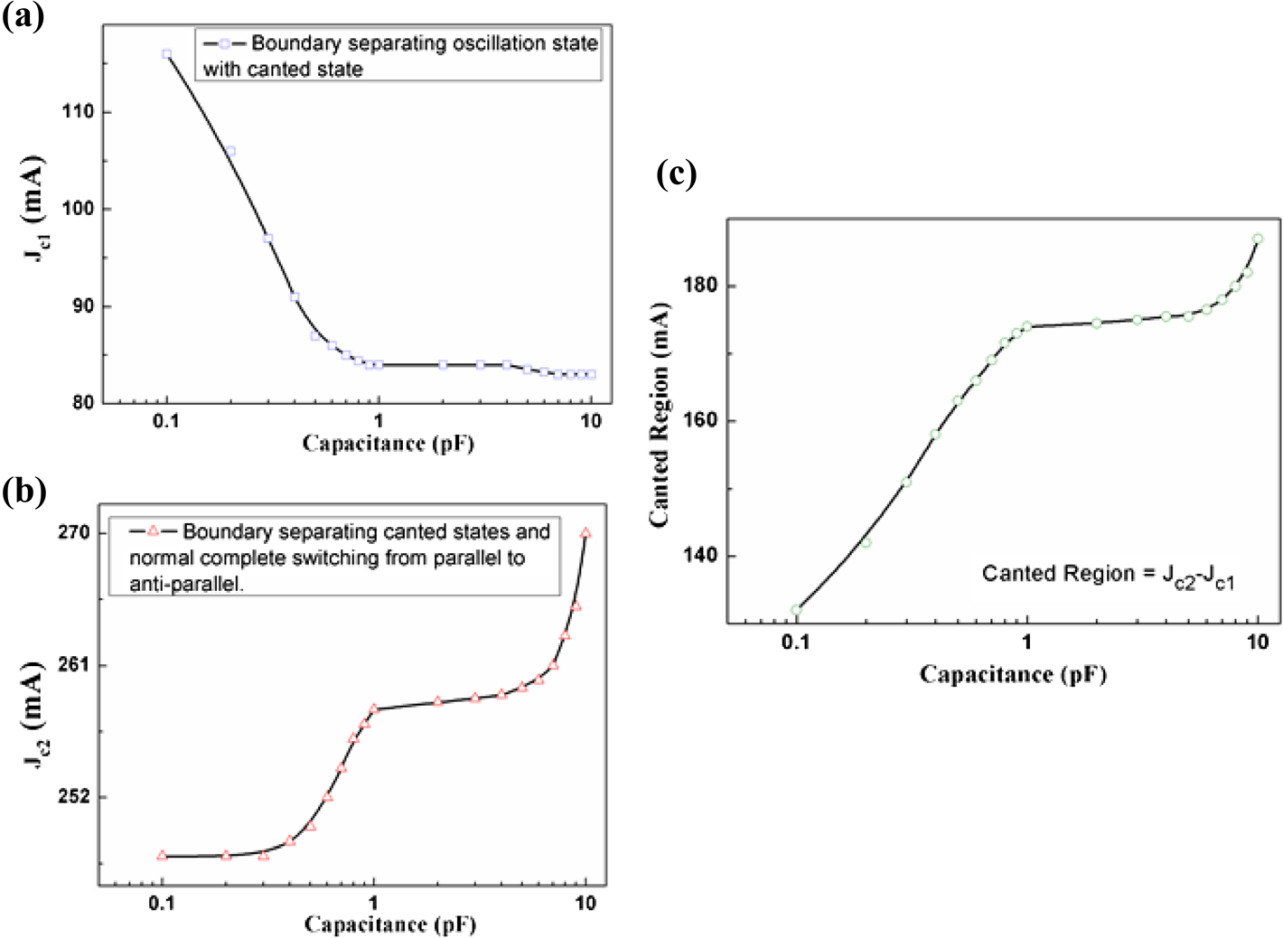
***J***_***c*1**_**, *J***_***c*2**_**, and canted region dependence on capacitance. (a)***J*_*c*1_(Boundary separating oscillation state with canted state) dependence on capacitance. **(b)***J*_*c*2_(Boundary separating canted states and normal complete switching from parallel to anti-parallel) dependence on capacitance. **(c)** Canted region (*J*_*c*2_ − *J*_*c*1_) dependence on capacitance.

The capacitance value is varied, and how the canted region evolves is explored in Figure 4. *J*_*c*1_ is defined as the current boundary separating oscillation state with canted state. As shown in Figure 4a, *J*_*c*1_ drastically decreases with capacitance in the range of 0.1 to 1 pF and tends to be stable with capacitance greater than 1 pF. J_c2_ is defined as the current boundary separating canted state and normal complete switching from parallel to anti-parallel. As shown in Figure 4b, *J*_*c*2_ increases with capacitance in a quasi-exponential tendency from 0.1 to 1 pF. This tendency is repeated for capacitance in the range of 1 to 10 pF. The difference between *J*_*c*2_ and *J*_*c*1_ results in the canted region as shown in Figure 4c. Obviously, the canted region maintains a positive correlation with the capacitance.

### C. Switching characteristics without external field

**Figure 5:**
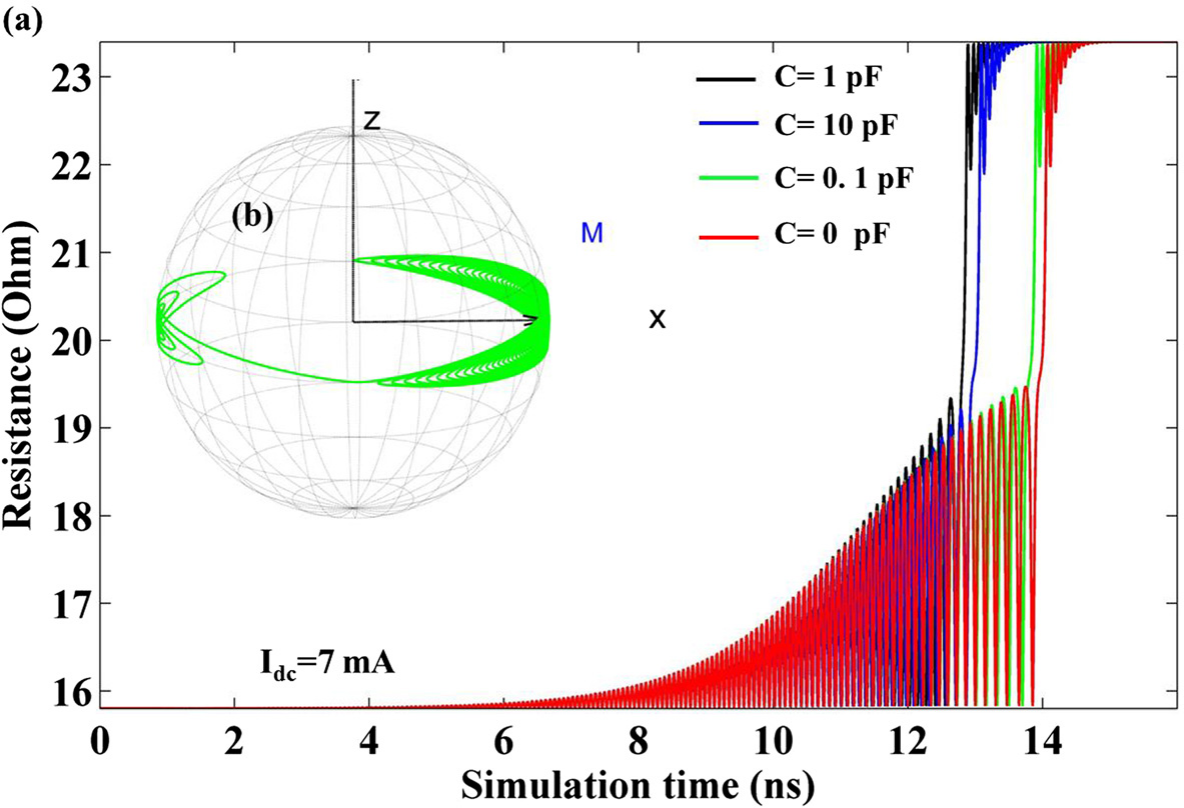
**When *I***_**dc**_ **= 7 mA. (a)** Time-evolved STO resistance for capacitance (C) =0, 0.1, 1, and 10 pF, respectively. **(b)** Free-layer magnetization switching trajectory for capacitance = 0.1 pF.

**Figure 6:**
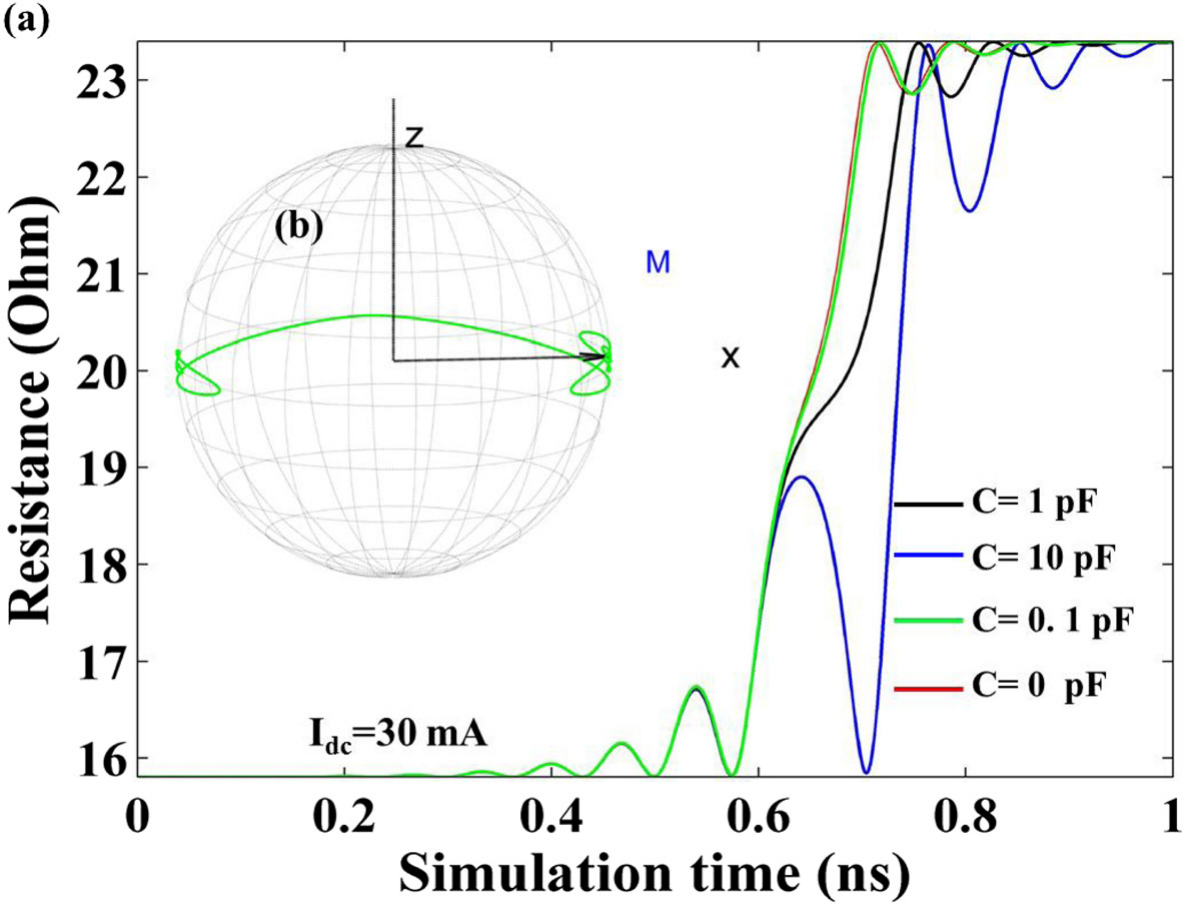
**When *I***_**dc **_**= 30 mA. (a)** Time-evolved STO resistance for C = 0, 0.1, 1, and 10 pF, respectively. **(b)** Free-layer magnetization switching trajectory for C = 0.1 pF.

**Figure 7:**
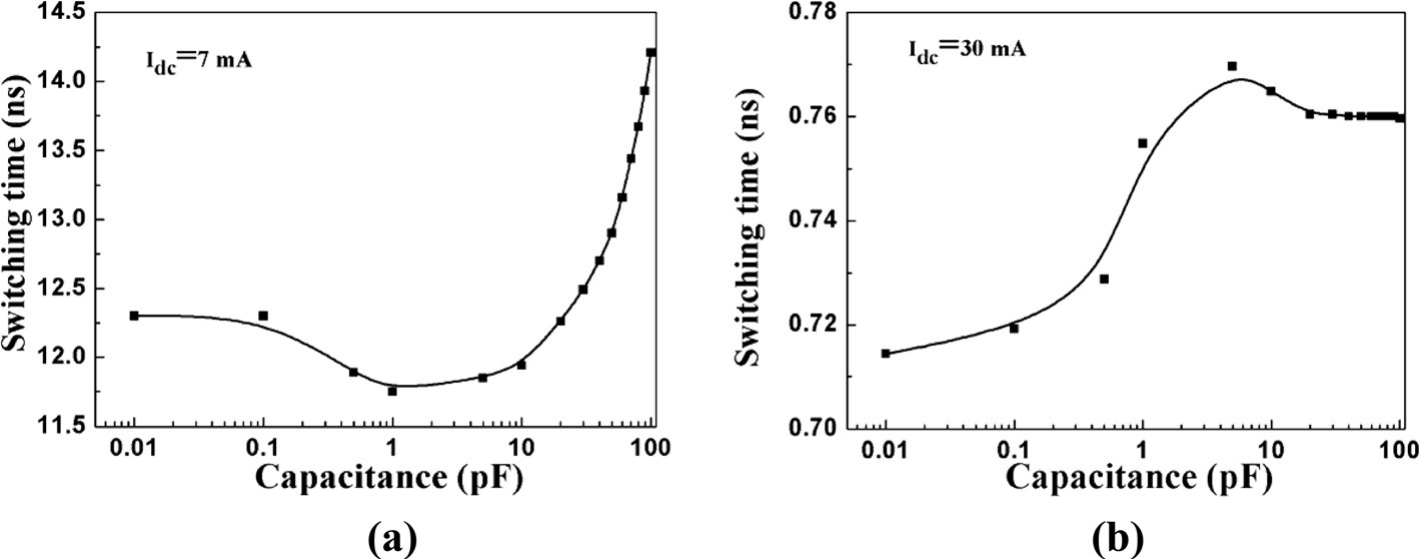
**STO free-layer magnetization switching time dependence on capacitance when (a)***I*_dc_ = 7 mA and **(b)***I*_dc_ = 30 mA.

Part A and Part B investigate the situation where the external field *H*_app_ is applied along the easy axis. In fact, for an in-plane magnetized STO in our system, the premises for a stable oscillation are the injected current and the external field. When the external field is absent, the injected current can only drive complete magnetization reversals from parallel state to anti-parallel. When a relatively small injected current (7 mA) is injected, the variation of STO resistance with the simulation time is shown in Figure 5a. It is found that capacitance can influence the free-layer magnetization switching time. Meanwhile, the trajectory in Figure 5b demonstrates that the existence of the capacitance renders plentiful unstable oscillating cycles before the final switching. It requires more oscillating cycles before final switching with increasing capacitance. When a relatively large injected current (30 mA) is injected, the variation of STO resistance with simulation time is shown in Figure 6. In this situation, the influence of capacitance on switching time is not obvious. The switching time is presented in Figure 7 with different capacitance values. The physical phenomena for STO free-layer switching time actually depends on three main factors: damping constant, in-plane spin torque component, and critical spin torque which intrigues the switching. In our study, we picked damping constant value 0.008, which is approach to the optimal value 0.013 for thin film switching. Thus we only consider the donation from the in-plane spin torque component and the critical spin torque which intrigues the switching. Based on the previous investigation [20], the switching time can be reasonably fitted by:

(11) $${t_{S}}^{-1}\propto a_{J}\left(c\right)-a_{\mathrm{crit}}\left(c\right)$$

where *a*_*J*_(c) represents the in-plane spin torque components with capacitance considered and a_crit_(*c*) represents the critical spin torque which intrigues the switching with capacitance considered. The reason the STO exhibits different dependences on the capacitance for different injected current is because when *I*_dc_ is relatively small (7 mA), the switching time is very slow since the in-plane spin torque component *a*_*J*_(*c*) (500 Oe in this case) has just exceed the value of the critical spin torque which intrigues the switching with capacitance considered a_crit_(*c*) (450 Oe in this case). However, when *I*_dc_ is relatively large (30 mA), the switching time is very fast since the in-plane spin torque component *a*_*J*_(*c*) has increased to level of 10,000 Oe, which far exceed a_crit_(*c*). Thus the switching time in Figure 7b is much smaller than the switching time in Figure 7a. On the other hand, when *I*_dc_ is relatively small (7 mA), the influence of capacitance on *a*_*J*_(*c*) is smaller than the influence of capacitance on a_crit_(*c*). When *I*_dc_ is relatively large (30 mA), the influence of capacitance on *a*_*J*_(*c*) is larger than the influence of capacitance on a_crit_(*c*). When *I*_dc_ is relatively small (7 mA), the switching time is mainly determined by a_crit_(*c*). However, the a_crit_(*c*) value is negatively correlated with the capacitance (calculation not presented here). Thus, for capacitance in the range of 0.01 to 1 pF, the a_crit_(*c*) value gradually decreases. For capacitance in the range of 1 to 100 pF, the a_crit_(*c*) value gradually increases. This explains the switching time tendency in Figure 7a. When *I*_dc_ is relatively large (30 mA), the switching time is mainly determined by *a*_*J*_(*c*). Since the *a*_*J*_(*c*) is very large and not influenced by the capacitance, the switching time only changes slightly (7.7%) as the capacitance increases.

## Conclusions

In summary, we have shown that with the external field applied, the STO oscillation frequency demonstrates a general negative correlation with the capacitance for injected current ranges from 8 to 12 mA while a general positive correlation with capacitance for injected current 20 mA. Canted regions are revealed for injected current higher than critical value. The free-layer magnetization switches from parallel state to canted state instead of from parallel state to anti-parallel state. When the external field is absent, the STO free-layer magnetization switching time exhibits two stages of variation with the capacitance for both small injected current value (7 mA) and large injected current value (30 mA). However, the variation trends are opposite for small injected current value (decrease in first stage and increase in second stage) and large injected current value (increase in first stage and decrease in second stage).

## Competing interests

The authors declare that they have no competing interests.

## Authors’ contributions

TZ and YZ designed the simulation model and implement it. TZ drafted the manuscript. All authors participated in the discussion of the results. All authors read and proved the final manuscript.
